# IL28B gene polymorphism rs12979860, but not rs8099917, contributes to the occurrence of chronic HCV infection in Uruguayan patients

**DOI:** 10.1186/s12985-018-0946-2

**Published:** 2018-03-02

**Authors:** Natalia Echeverría, Daniela Chiodi, Pablo López, Adriana Sanchez Ciceron, Jenniffer Angulo, Marcelo López-Lastra, Paola Silvera, Adrian Canavesi, Carla Bianchi, Valentina Colistro, Juan Cristina, Nelia Hernandez, Pilar Moreno

**Affiliations:** 1 Laboratorio de Virología Molecular, Centro de Investigaciones Nucleares, Facultad de Ciencias, Universidad de la República, 2055 Montevideo, Mataojo Uruguay; 2 Clínica de Gastroenterología, Hospital de Clínicas, Facultad de Medicina, Universidad de la República, 11600 Montevideo, Uruguay; 3 Departamento de Laboratorio Clínico, Hospital de Clínicas, Facultad de Medicina, Universidad de la República, Montevideo, Uruguay; 4 Laboratorio de Virología Molecular, Instituto Milenio de Inmunología e Inmunoterapia, Centro de Investigaciones Médicas, Pontificia Universidad Católica de Chile, Santiago, Chile; 5 Sanatorio SEMM-Mautone, Maldonado, Uruguay; 6 Departamento de Genética, Facultad de Medicina, Universidad de la República, Montevideo, Uruguay

**Keywords:** rs12979860, rs8099917, Hepatitis C, Genotypic distribution

## Abstract

**Background:**

Host single-nucleotide polymorphisms (SNPs) near the interleukin 28B (*IL28B*) locus are associated with sustained virological response to antiviral therapy and with spontaneous Hepatitis C Virus (HCV) clearance. Prevalence of these SNPs varies depending on ethnicity. The impact of IL28B SNPs in HCV-infected patients is currently unknown in Uruguay. Therefore, the aim of this study was to evaluate and compare the distribution of polymorphisms in the IL28B gene (rs12979860 and rs8099917) among HCV-infected patients and healthy individuals in Uruguay and thus assess their possible association with the establishment of HCV infection.

**Methods:**

DNA was recovered from 92 non-infected individuals and 78 HCV-infected patients and SNPs were determined by RFLP and allelic discrimination by real-time PCR.

**Results:**

The distribution of rs12979860 genotypes for the infected population was 29.5%-CC, 47.4%-CT and 23.1%-TT and for the control group 45.7%, 42.4% and 11.9%, respectively. Prevalence in both infected and uninfected individuals is similar to that reported in other countries with admixed populations. The distribution of rs8099917 genotypes for the infected population was 57.7%-TT, 27.2%-TG and 14.1%-GG and for the control group 60.9%, 33.7% and 5.4%, respectively. The comparison of rs12979860 genotype distribution between the two populations evidenced a higher prevalence of the favourable genotype (CC) in the uninfected control group (*p* < 0.05). Additionally, results generated using logistic regression analysis show that individuals carrying rs12979860-TT or CT genotypes have a higher likelihood of developing chronic hepatitis upon infection with HCV, when compared to CC carriers, considering rs8099917 genotype as constant.

**Conclusion:**

Patients with HCV infection have a statistically significant lower prevalence of the favourable rs12979860 genotype when compared to uninfected individuals; therefore we can establish that only IL28B rs12979860-CT and TT genotypes seem to contribute to the occurrence of chronic HCV infection in the cohort of Uruguayan population studied. Considering that a trend towards a higher frequency of “good” response genotypes was observed in responder patients, we believe that IL28B rs12979860 genotyping could be a useful tool for predicting different therapies outcome, including in the DAA era.

**Electronic supplementary material:**

The online version of this article (10.1186/s12985-018-0946-2) contains supplementary material, which is available to authorized users.

## Background

With an estimate of 71 million infected individuals worldwide, Hepatitis C virus (HCV) represents a major health problem and is currently the leading cause of cirrhosis, hepatocellular carcinoma and an indication for liver transplantation worldwide [1]. Despite the recent development of highly effective compounds, direct-acting agents (DAAs), designed to specifically block HCV replication, the combination of pegylated interferon alpha (peg-IFN-α) plus ribavirin (RBV) is still the main option for HCV treatment in several countries, including Uruguay. This therapy, however, only yields a sustained virological response (SVR) in about half of the treated patients when infected with HCV genotype 1; the most prevalent genotype in Uruguay [2–4]. The poor response to treatment, its high cost, as well as the frequent occurrence of severe side effects associated to the use of peg-IFN-α/RBV [5], highlight the relevance of enabling the means for predicting the patient’s response to antiviral therapy. This would allow an early selection of the most adequate cost-effective HCV treatment for each particular patient. Different predictors of SVR are known, some of which are linked to the virus (genotype, viral load) whereas others are linked to the host (age, sex, race, liver fibrosis, genetic factors) [6].

Among the host factors known to be associated with the outcome of HCV-treatment, specific single-nucleotide polymorphisms (SNPs) located near the interleukin 28B (IL28B) gene (which codes for IFN-λ-3) have shown a significant relationship with both spontaneous virus clearance and response to peg-IFN-α/RBV treatment [7–10]. Patients carrying the “good” response genotypes (major allele in homozygosity) are more likely to resolve the infection than those carrying the “poor” response genotypes (risk allele in homozygosity or heterozygosity) [7–9]. Since its characterization, IL28B genotyping has proven useful in guiding clinicians towards the selection of the most adequate patient-personalized therapy [11]. Nevertheless the strong predictive value of these SNPs is only applicable for patients infected with HCV genotypes 1 and 4 [12, 13]. In cases of infection with genotypes 2 or 3 IL28B SNP information is valuable only for patients with detectable levels of HCV RNA at week 4 (absence of rapid virological response) [14]. Interestingly, IL28B genotyping has also proven relevant to anticipate HCV SVR in regimens using DAAs either in combination with IFN [15–19] or in its absence [20].

Ethnicity is also a host factor that correlates with the patient’s ability to respond to antiviral treatment [21, 22]. This phenomenon has been partly explained by the prevalence of IL28B polymorphisms within different ethnic groups [23]. For example, the major C allele (“good” response allele) of rs12979860 SNP in the general population has a frequency of 0.23–0.55 among Africans, 0.53–0.80 among Europeans and 0.66–1.00 among Asians [24]. In this context, Asians and Europeans are better responders to peg-IFN-α/RBV treatment than African-descendants [22]. The case for Latin America is less clear as the prevalence of these IL28B SNPs is known only in a few countries [25–31]. Furthermore, given the admixed genetic background of the Latin American populations, it is unreasonable to assume an equivalent distribution of IL28B SNPs in all South America. In fact, when comparing different populations from Latin America a sharp difference in allele frequencies for IL28B is observed [24]. For example, the C allele is less prevalent in Mexico (0.38–0.56) than in Brazil (0.64–0.824) [24]. To date no genetic information regarding IL28B SNP prevalence is available for the Uruguayan population. Noteworthy, the Uruguayan population exhibits a European, Amerindian, and African contribution to ancestry, being the Europeans the main contributors [32]. The contribution of Amerindians and Africans to the Uruguayan ancestry largely varies throughout the country stressing the heterogeneity of this South American population [32].

Hence, the aim of this study was to determine the prevalence of IL28B polymorphisms (rs12979860 C > T and rs8099917 T > G) in treatment-naïve HCV-infected patients using uninfected individuals as controls and thus assess their possible association with the establishment of HCV infection. The results suggest that in Uruguay the prevalence of the favourable rs12979860 genotype (CC) is higher in the control group (*p* < 0.05). Additionally, considering rs8099917 genotype constant, individuals carrying rs12979860-TT or CT genotypes have a higher likelihood of developing chronic hepatitis upon infection with HCV, when compared to CC carriers.

## Methods

### Study population

A cross-sectional and observational study was conducted in Uruguayan individuals with and without HCV infection, recruited between 2014 and 2015 at the Gastroenterology Clinic from Hospital de Clínicas (Control group, *n* = 92; HCV-infected group, *n* = 78). Chronic HCV-infected patients were treatment naïve and over 18 years old. All samples analysed in this study were negative for hBsAg and HIV and in the case of the control group, all individuals showed absence of reactive serology for HCV. The information obtained from the study populations included sex and age. For the HCV-infected patients virus genotype and stage of liver disease were included, if previously determined (See Table 1). Additionally, 48 of the 78 enrolled HCV-infected patients underwent dual therapy (peg-IFN-α plus RBV) during the study, 42 of which had completed it by the time of writing. SVR was defined as absence HCV RNA in serum 24 weeks after the end of treatment. Non-response (NR) to therapy was defined as HCV viral load decline less than 2 logs at week 12 during therapy or detectable serum HCV RNA at any other time during therapy. Relapse (R) was defined as undetectable level of HCV RNA by the end of treatment which becomes detectable after discontinuation of therapy. For analyses, we divided patients into two groups: patients who achieved SVR and those who did not (NR and R).

**Table 1:** Demographic characteristics of both populations studied

Variable	Total population (*n* = 170)	HCV-infected population (*n* = 78)	Uninfected population (*n* = 92)	Statistical test and value	*p* value
*Gender*					
Male (%)	115 (67.6)	48 (61.5)	67 (72.8)	χ^2^ 2.457	0.117
Female (%)	55 (32.4)	30 (38.5)	25 (27.2)		
*Age (years), (mean ± SD)*	42.7 ± 12.6	46.3 ± 13.1	40 ± 11.4	t = 3.230	0.002*
*Genotype (n = 78), n (%)*					
G1	45 (57.7)				
G2	3 (3.8)				
G3	12 (15.4)				
Not determined	18 (23.1)				
*Liver Stage (n = 49), n (%)*					
1	10 (20.4)				
2	10 (20.4)				
3	5 (10.2)				
4	24 (49.0)				

χ^2^ Chi-square test; t: Student’s t-test; *p < 0.05

### Ethical and regulatory considerations

The study was conducted according to national and international ethical guidelines (good clinical practice, Nuremberg statements, Declaration of Helsinki) and local regulatory rules (Mercosur Standards/GMC/RES No. 129/96). The protocol was approved by the Ethics Committee of the Hospital de Clínicas on October 24th, 2014 and all patients gave written informed consent. Access to personal information was restricted to the medical doctors. The genetic information extracted from the samples was used exclusively for the purposes of this study.

### Genotyping of SNPs rs12979860 and rs8099917

Genomic DNA was extracted from peripheral white blood cells by QIAamp DNA Kit (QIAGEN). All samples from HCV-infected individuals were SNP genotyped by end-point PCR amplification following restriction fragment length polymorphism analysis as previously described [33]. For rs12979860 primers used were: rs12-F 5’-GCGGAAGGAGCAGTTGCGCT-3′ (sense) and bst-R 5’-GGGGCTTTGCTGGGGGAGTG-3′ (antisense). For rs8099917 primers used were: rs80-F 5’-CCCACTTCTGGAACAAATCGTCCC-3′ (sense) and rs80-R 5’-TCTCCTCCCCAAGTCAGGCAACC-3′ (antisense) [33]. All samples from the control group were genotyped by real-time PCR using TaqMan SNP allelic discrimination assays carried out using StepOne Real-Time PCR System (Applied Biosystems). The primers, MGB probes and conditions used to amplify rs12979860 have been previously described [27]. The rs8099917 SNP genotyping was determined by a TaqMan® Pre-designed SNP Assay (Applied Biosystems) (AB) reference: C_11710096_10. Genotyping of each sample was attributed automatically by the StepOne Software v2.2.2. Positive and negative controls (previously verified by direct Sanger sequencing) were used in each genotyping assay.

### Statistical analyses

Data are presented as percentages (categorical variables), means and standard deviations (continuous variables). Comparisons between the two groups were made using the chi-square (*χ*^2^) test or Fisher’s exact test (when cell sample sizes were less than five) for categorical variables and Student’s t-test for continuous variables. The existence of differences in genotypic frequencies between groups was assessed by Chi-square test under the three main inheritance models: codominant, dominant and recessive. A Monte Carlo permutation method was used for multiple test correction. A *p* value less than 0.05 was considered statistically significant. Raw *p* values are shown in the tables. Possible deviations from Hardy-Weinberg equilibrium were studied in the control population (both exact and Chi-squared tests were performed) using PLINK Package version 1.9 (http://zzz.bwh.harvard.edu/plink/) [34]. We performed univariable and multivariable logistic regression analyses in order to analyse the independent contribution of each polymorphism to the occurrence of chronic HCV infection (dependent or outcome variable). The categorical independent variables included in the full multivariable model were rs12979860 and rs8099917 SNP genotypes (CC, CT, TT and TT, TG, GG, respectively) adjusted by age and gender. An interaction multiplicative term between both SNPs was also tested in order to consider their joint effect, if any. Odds Ratios (OR) and 95% confidence intervals were calculated. The analyses were performed using the IBM® SPSS® Statistics version 23 Software and R software version 3.4.0 [35]. Linkage disequilibrium analysis was performed using Haploview Software [36].

## Results

### Demographic characteristics of infected patients and controls

Seventy-eight treatment-naïve patients with chronic HCV infection were recruited. 61.5% were male with an average age of 46 years. In all cases, chronic infection was confirmed by a viral load above the limit of detection or by qualitative PCR detection. HCV genotypic analysis confirmed the prevalence of genotype 1 (57.7%) in the cohort. The control group included 92 individuals, all of which were HCV, HIV and HBV negative by serologic methods. 72.8% were male with an average age of 40 years. All demographic characteristics are presented in Table 1, including the histological stages for the infected population and the viral genotypes, if previously determined. No significant difference was found in terms of gender distribution (*p* ≥ 0.05). On the contrary, the difference in age between groups was statistically significant (*p* < 0.05). The stage of liver disease according to Metavir score was available in 49 of the 78 infected patients. Cirrhosis (stage 3 and 4) was observed in 29 (59.2%) of them (Table 1).

### SNPs rs12979860 and rs8099917 genotype distribution in non-infected individuals and in HCV-infected patients

**Fig. 1:**
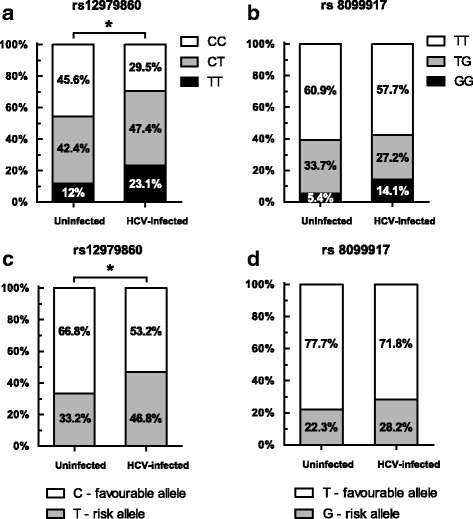
IL28B polymorphisms in a Uruguayan HCV-infected population (*n* = 78) and a Uruguayan uninfected population (*n* = 92). **a** SNP rs12979860 genotype distribution, where CC is considered as the good genotype and CT/TT as the unfavourable genotypes. The difference between groups is statistically significant (χ^2^ test - codominant model, *p* = 0.045). **b** SNP rs8099917 genotype distribution, where TT is considered as the good genotype and TG/GG as the unfavourable genotypes. **c** SNP rs12979860 allelic distribution. The distribution between both populations is statistically significant (χ^2^ test, *p* = 0.010). **d** SNP rs8099917 allelic distribution

Several reports establish a strong link between SNPs rs12979860 and rs8099917 with the response to peg-IFN-α/RBV treatment for HCV genotype 1 infected patients [7, 9, 13, 25, 28, 29, 31, 37–39]. To date no study exists that shows the distribution of these polymorphisms within the Uruguayan population despite the fact that HCV genotype 1 is confirmed as the most prevalent viral genotype in the country (Table 1) [40]. To gain information regarding the distribution of IL28B SNPs among the Uruguayan population the genomic DNA from 92 non-infected, non-related individuals were analysed. The distribution of rs12979860 genotypes within this control group were 45.7% CC, 42.4% CT and 11.9% TT, while for the rs8099917 they were 60.9% TT, 33.7% GT and 5.4% GG (Fig. 1 and Table 2). SNP rs12979860 and rs8099917 showed to be in Hardy-Weinberg equilibrium (χ^2^ tests, *p ≥* 0.05) in the control group, which allowed us to proceed with the genotype distribution comparison in both populations. Next we sought to establish the distribution of IL28B polymorphisms within a Uruguayan cohort of 78 treated naïve HCV-infected patients. SNP analysis in the HCV-infected cohort revealed that the frequencies for the rs12979860 genotypes were 29.5% CC, 47.4% CT and 23.1% TT, while frequencies for the rs8099917 TT, GT and GG genotypes were 57.7%, 28.2% and 14.1%, respectively (Fig. 1 and Table 2). Under both a codominant as well as a dominant model, the genotype distribution corresponding to rs12979860 (CC, CT, TT) between the non-infected control group and the HCV-infected cohort (Fig. 1a) evidenced to be statistically significant (*p*˂0.05) even after multiple test correction, with a higher prevalence of the favourable genotype (CC) within the control group (Table 2). A similar analysis was done for rs8099917 genotype distributions; however no statistically significant differences between both cohorts were found (Fig. 1b and Table 2). The allelic frequencies between both populations also showed to be statistically different only for rs12979860 (Fig. 1c and 1d). No association was observed between IL28B genotype and other variables such as gender, HCV genotype or liver fibrosis stage (Additional files 1 and 2).

**Table 2:** Genotype frequency of SNP rs12979860 and rs8099917 in both populations

IL28B SNP	HCV-infected population (*n* = 78)	Uninfected population (*n* = 92)	Statistical test and value	*p* value
*rs12979860, n (%)*			χ^2^_cd_ 6.185	0.045*
CC	23 (29.5)	42 (45.7)		
CT	37 (47.4)	39 (42.4)		
TT	18 (23.1)	11 (11.9)		
CT + TT d	55 (70.5)	50 (54.3)	χ^2^_d_ 4.671	0.031*
CC + CT r	60 (76.9)	81 (88.0)	χ^2^_r_ 3.689	0.055
*rs 8,099,917, n(%)*			χ^2^_cd_ 3.850	0.146
TT	45 (57.7)	56 (60.9)		
TG	22 (28.2)	31 (33.7)		
GG	11 (14.1)	5 (5.4)		
TG + GG d	33 (42.3)	36 (39.1)	χ^2^_d_ 0.177	0.674
TT + TG r	67 (85.9)	87 (94.6)	χ^2^_r_ 3.720	0.054

χ^2^ Chi-square test; cd = Codominant model; d = Dominant model; r = Recessive model; *p < 0.05

Of the 42 HCV-infected patients who underwent and finished IFN-α/RBV treatment, 17 achieved SVR, whereas 25 did not (3 NR and 21 relapsers). Among those who responded favourably, 23.5% carried the rs12979860 good-response genotype (CC) while 58.8% carried the rs8099917 favourable genotype (TT). Among non-responder and relapser patients, these frequencies were lower (20.0% - CC and 48.0% - TT, respectively). Nevertheless, chi-squared and Fischer’s exact tests did not evidence statistically significant differences in distributions of IL28B genotypes between both groups.

Next we sought to determine the contribution of IL28B SNPs to the occurrence of chronic HCV-infection by performing logistic regression analyses (Table 3). The results of univariable and multivariable (adjusted by age and sex) models suggest that when rs8099917 genotype is constant, individuals carrying rs12979860 unfavourable genotypes TT and CT on average have a 5.783 (TT) and 3.086 (CT) fold higher likelihood (adjusted OR_TT_ = 5.783, *p* = 0.023; adjusted OR_CT_ = 3.086, *p* = 0.015) of developing a chronic infection upon infection with HCV when compared to individuals hosting the favourable genotype (CC). Noteworthy, all individuals homozygous for the favourable allele of rs12979860 (CC) also carried the favourable genotype of rs8099917 (TT). It should be noted that no interaction between the SNPs was evidenced, as revealed by a non-converging model. In addition, rs12979860 – TT/CT as well as age were found to be associated with a higher chance of chronic infection occurrence in both univariable and multivariable models.

**Table 3:** Logistic regression of each polymorphism in cases (chronic HCV-infected patients) versus controls (uninfected individuals)

Factor	Univariable Models		Multivariable Model			
	Estimate	OR (95% CI)	*p* value	Estimate	OR (95% CI)	*p* value
*rs12979860*						
CC	NA	1		NA	1	
TT	1.095	2.988 (1.208–7.395)	0.018*	1.755	5.783 (1.279–26.142)	0.023*
TC	0.550	1.732 (0.879–3.415)	0.113	1.127	3.086 (1.247–7.642)	0.015*
*rs 8,099,917*						
TT	NA	1		NA	1	
GG	1.007	2.738 (0.886–8.454)	0.080	−0.240	0.787 (0.141–4.375)	0.784
TG	−0.124	0.883 (0.451–1.730)	0.717	−0.672	0.511 (0.197–1.325)	0.167
*Age*	0.051	1.052 (1.023–1.082)	<0.001*	0.036	1.037 (1.008–1.067)	0.012*
*Gender*	−0.516	0.597 (0.312–1.141)	0.118	0.546	1.727 (0.843–3.537)	0.135

Odds ratio were constructed considering favourable genotypes (rs12979860-CC and rs 8,099,917-TT) as reference. OR: Odds ratio; CI: Confidence Interval; *p < 0.05

The results of the genotype distribution comparisons as well as the logistic regression are supported by Haploview analyses showing that both SNPs are in weak Linkage Disequilibrium in both of the studied populations (controls: r^2^ = 0.57; infected-patients: r^2^ = 0.37; both: r^2^ = 0.48). This observation also indicates that for the studied population of HCV-infected patients the association between IL28B SNPs and development of HCV chronicity is primarily driven by only one of the evaluated polymorphisms.

## Discussion

Infections with HCV have become a major cause of liver cancer and one of the most common indications for liver transplantation [1]. In Uruguay the combination of peg-IFN-α plus RBV is still the main option for HCV treatment, despite the fact that HCV genotype 1 is the predominant genotype among the infected patients in Uruguay [40]. This, in addition to the high cost of the new direct-acting therapies, highlight the relevance of searching for new indicators of response to antiviral therapy in the Uruguayan population in order to provide information that could be a useful guide for clinicians, enabling them to select a more patient-personalised anti-HCV therapy.

Many host factors have been associated with HCV-treatment outcome, among which, specific single-nucleotide polymorphisms (SNPs) located near the interleukin 28B (IL28B) gene have been shown to exhibit a significant relationship with both spontaneous virus clearance and response to peg-IFN-α/RBV treatment [7–10]. In this context, and considering that no information on IL28B SNPs genotype distribution was known for Uruguay, we were interested in evaluating the frequency of IL28B rs12979860 and rs8099917 in a cohort of Uruguayan individuals (HCV-infected as well as uninfected). With this aim, 92 healthy individuals and 78 HCV-infected patients were studied.

In full agreement with what others have reported [26, 27, 30, 41–43], the frequency of the IL28B rs12979860 favourable genotype (CC), which strongly predicts spontaneous clearance of HCV infection, was less prevalent among the studied HCV-infected population than within the non-infected individuals. This differential genotypic distribution was statistically significant (*p*˂0.05) (Fig. 1). This bias toward the less favourable genotype for rs12979860 is most probably associated to the way HCV-infected patients were selected for the study as all were recruited in a hospital setting. No significant differences between the frequencies of the protective rs8099917-TT genotype in general population and the studied HCV-infected patients were observed. The unfavourable homozygous genotypes were found at a low frequency in both populations under study (rs12979860-TT 23.1% vs 11.9% and rs8099917-GG 14.1% vs 5.4%, in the infected and control group, respectively). Given that a higher prevalence of favourable rs12979860 genotype has been found among healthy individuals when compared to HCV-infected patients [41], it is plausible that our results support the notion of a protective effect of IL28B “good” response genotype within the Uruguayan population, as has been previously suggested by others [10, 44].

Additionally, by means of logistic regression analyses we show that individuals carrying rs12979860-TT or CT genotypes have a higher likelihood of developing chronic hepatitis upon infection with HCV, when compared to CC carriers, when the rs8099917 genotype is constant (Table 3). Therefore, our results suggest that within the Uruguayan population rs12979860 might be a better predictor than rs8099917, at least in terms of occurrence of chronic HCV infection. This is also in agreement with the fact that these SNPs seem to be in weak linkage disequilibrium, which indicates that only one of them might be associated with development of chronicity in the studied Uruguayan cohort.

An interesting finding of this work is that it shows that the genotype frequencies in Uruguay seem to fall in the same ranges as those found in other countries (Table 4), this with the exception of Asian countries where the prevalence of protective genotypes were found to be higher [37, 45]. Although the allelic frequencies of both SNPs in the control group fall in the range of the frequencies reported for different European populations [24], the distribution of genotypes in the Uruguayan population seems to be more similar to the frequencies reported in Brazil [30, 31] and the rs12979860-CC prevalence among infected patients (29.5%) resembles the one reported for Hispanics, rather than Caucasians (Table 5). This observation raises important questions regarding the Uruguayan genetic background and ethnicity. Uruguay has no Native American or African-descendant communities and until the 1980s its national identity was regarded as almost strictly Caucasian [32]. More recently, however, several reports based both on classical markers as well as nuclear DNA analysis have revealed that the Uruguayan population has a small but important African and Native American ancestry contribution (see [32] for a detailed review). Thus, it is tempting to speculate that it is this previously non-considered ethnic contribution that would explains why the SNPs frequencies within the Uruguayan population closely resemble those of admixed rather than a Caucasian population.

**Table 4:** Il28B favourable genotype prevalence reported in different countries

IL28B SNP	Uninfected population % (*n*)	HCV-infected population % (*n*)	Country	Year of publication
*Rs12979860; CC*				
	44.7 (378)	45.6 (283)	Spain	2010 [42]
	45.1 (122)	26.9 (108)	Egypt	2015 [43]
	44.6 (92)	32.1 (136)	Turkey	2015 [41]
	43.7 (142)	38.0 (921) – 42.0 (100)	Iran	2012 [49], 2016 [50]
	86.9 (320)	88.6 (297)	China	2015 [51]
	ND	24.1 (83)	Mexico	2012 [25]
	35.7 (185)	ND	Bolivia	2014 [26]
	38.1 (76)	ND	Peru	2014 [26]
	38.8 (98)	ND	Paraguay	2014 [26]
	37.0 (405)	20.2 (99)	Chile	2013 [27], 2011 [28]
	51.6 (991)	18.4 (102)	Argentina	2014 [26], 2011 [29]
	47.4 (190)	24.0 (221) – 30.9 (175)	Brazil	2012 [31], 2015 [30]
	45.6 (92)	29.5 (78)	Uruguay	This study
*rs8099917; TT*				
	64.1 (142)	58.3 (921)	Iran	2012 [49]
	88.7 (320)	89.6 (297)	China	2015 [51]
	89.3 (197)	81.0 (400)	Taiwan	2016 [45]
	ND	27.5 (80)	Mexico	2012 [25]
	46.9 (405)	29.3 (99)	Chile	2013 [27], 2011 [28]
	ND	40.2 (102)	Argentina	2011 [29]
	67.8 (199)	54.2 (177) – 63.1 (222)	Brazil	2015 [30], 2012 [31]
	60.9 (92)	57.7 (78)	Uruguay	This study

**Table 5:** Rs12979860 favourable genotype (CC) prevalence reported according to ethnicity in HCV-infected patients

HCV-infected population % (*n*)	Ethnicity	Year of publication
33.3 (2582) – 37.2 (1171)	Caucasians	2016 [52], 2010 [13]
22.9 (105) – 29.3 (116)	Hispanics	2016 [52], 2010 [13]
10.4 (48) – 14.0 (300)	African Americans/Black	2016 [52], 2010 [13]
49.2 (181)	Asian	2016 [52]

Several reports from different countries and regions have found associations between favourable genotypes of IL28B SNPs and SVR [8, 25, 28, 29, 31, 37, 38, 46], regretfully we did not find evidence which supports this link in our Uruguayan population. Differences in SNPs genotype distributions between SVR and NR/R patients were not statistically significant (chi-squared and Fisher’s exact tests considering the three modes of inheritance: codominant, dominant and recessive). This might be attributable to the small number of patients included in this study that had finished their therapy by the time of writing this report (*n* = 42) which accounts for 53.8% of all HCV-infected patients enrolled. In addition, of those 42 HCV-infected individuals that had completed their dual treatment, 17 achieved SVR while 25 were either non-responders or relapsers (NR/R). Of those who achieved SVR, 23.5% (n = 4/17) harboured the favourable IL28B genotype (rs12979860-CC), whereas only 20.0% of the NR/R patients had the favourable genotype (*n* = 5/25). These results suggest a slight trend towards a higher frequency of “good” response genotypes in responder patients. This tendency has also been observed in other populations in Latin America where the frequency of CC-carriers among the NR patients (2% in Chile [28] and 20% in Mexico [25]) seems to be lower than among SVR patients. The small number of patients corresponding to each group (SVR or NR/R) might explain why we were not able to confirm the association between IL28B genotypes and response to therapy. Despite Heo et al. (2014) [47] also reported no association even when including a larger number of patients (*n* = 156), the ethnic disparities between Uruguayan and Korean populations does not allow us to confirm our findings. A limitation of our analyses, however, is that due to the limited number of patients, association studies were performed without taking into account the viral genotype. In this respect, some authors report no association between IL28B SNPs and SVR in patients infected with HCV genotype 3 [48]. This fact might have biased our results since we included both HCV genotype 1 and 3 infected patients.

As mentioned before, despite the fact that some of the new treatment regimens including DAAs have been approved for use by Uruguayan national authorities, their costs are not always covered by our health system, making them unaffordable for most of the population. Therefore, we believe it would still be relevant for our country to use hosts genotype data as a predictor of response to HCV-treatment. Furthermore, IL28B genotype seems to be also informative when new DAAs are used, both in the context of protease inhibitors combination therapy [15–19] as well as in IFN-free regimens with sofosbuvir (polymerase inhibitor) and ledipasvir (NS5A inhibitor) [20]. Therefore, it is feasible to propose that IL28B genotyping could be a powerful tool use in Uruguay to predict the best personalised anti HCV-treatment in the upcoming years.

## Conclusions

In conclusion, the present study shows that the favourable genotypes rs12979860-CC and rs8099917-TT were present in 29.5% and 57.7% of the Uruguayan population infected with HCV, respectively. As expected, the prevalence within the non-infected population was higher (45.6% for rs12979860 and 60.8% for rs8099917). Only IL28B rs12979860-CT and TT genotypes seem to contribute to the occurrence of chronic HCV infection in the cohort of Uruguayan population studied. Considering that a slight trend towards a higher frequency of “good” response genotypes was observed in responder patients, we believe that IL28B rs12979860 genotyping could be a useful tool for predicting different therapies outcome, including in the DAA era. It is worth mentioning that this study also found that allele and genotype frequencies closely resemble those of an admixed population rather than a uniformly European-descendant one, which is in agreement with previous studies on non-Caucasian ancestry contribution to Uruguayan population [32].

## Additional files

Additional file 1: SNP rs12979860 genotypes according to infected patient characteristics. (DOCX 15 kb)

Additional file 2: SNP rs8099917 genotypes according to infected patient characteristics. (DOCX 15 kb)

## Abbreviations

DAA: Direct-acting Agent
HCV: Hepatitis C Virus
IL28B: Interleuquin 28B
Peg-IFN-α: pegylated interferon alpha
RBV: Ribavirin
SNP: Single-nucleotide Polymorphism
SVR: Sustained Virological Response
