# Supplementary material for: IL28B gene polymorphism rs12979860, but not rs8099917, contributes to the occurrence of chronic HCV infection in Uruguayan patients
Source: Virol J. 2018 Mar 2;15:40. doi: 10.1186/s12985-018-0946-2 (PMC5833045; doi:10.1186/s12985-018-0946-2)
Supplement: Supplementary file 1 — SNP rs12979860 genotypes according to infected patient characteristics. (DOCX 15 kb) [file 12985_2018_946_MOESM1_ESM.docx]

**Additional File 1.** SNP rs12979860 genotypes according to infected patient characteristics

| Variable | CC (*n*=23) | CT (*n*=37) | | TT (*n*=18) | Statistical test and value | *p* values |
| --- | --- | --- | --- | --- | --- | --- |
| *Gender, n (n=78)* | | | | | χ^2^  0.689 | 0.709 |
| Male | 15 | 21 | 12 | |  | |
| Female | 8 | 16 | 6 | |  | |
| *HCV Genotype, n (n= 60)* | | | | | χ^2^  3.912 | 0.42 |
| G1 | 14 | 22 | 9 | |  | |
| G2 | 1 | 1 | 1 | |  | |
| G3 | 1 | 6 | 5 | |  | |
| *Liver stage, n (n=49)* | | | | | χ^2^  5.014 | 0.54 |
| 1 | 2 | 7 | 1 | |  | |
| 2 | 2 | 6 | 2 | |  | |
| 3 | 0 | 4 | 1 | |  | |
| 4 | 9 | 10 | 5 | |  | |

χ^2^ Chi-square test
