# Supplementary material for: IL28B gene polymorphism rs12979860, but not rs8099917, contributes to the occurrence of chronic HCV infection in Uruguayan patients
Source: Virol J. 2018 Mar 2;15:40. doi: 10.1186/s12985-018-0946-2 (PMC5833045; doi:10.1186/s12985-018-0946-2)
Supplement: Supplementary file 2 — SNP rs8099917 genotypes according to infected patient characteristics. (DOCX 15 kb) [file 12985_2018_946_MOESM2_ESM.docx]

**Additional File 2.** SNP rs8099917 genotypes according to infected patient characteristics

| Variable | TT  (*n*=45) | GT  (*n*=22) | | GG  (*n*=11) | Statistical test and value | *P* values |
| --- | --- | --- | --- | --- | --- | --- |
| *Gender* | |  | | | χ^2^  0.892 | 0.64 |
| Male (%) | 26 | 14 | 8 | |  | |
| Female (%) | 19 | 8 | 3 | |  | |
| *Genotype, n (n=60)* | |  | | | χ^2^ 0.698 | 0.95 |
| G1 | 25 | 14 | 6 | |  | |
| G2 | 2 | 1 | 0 | |  | |
| G3 | 7 | 3 | 2 | |  | |
| *Liver stage, n (n=49)* | |  | | | χ^2^ 4.239 | 0.64 |
| 1 | 5 | 5 | 0 | |  | |
| 2 | 5 | 3 | 2 | |  | |
| 3 | 3 | 2 | 0 | |  | |
| 4 | 15 | 7 | 2 | |  | |

χ^2^ Chi-square test
